# Identifying a Biocontrol Bacterium with Disease-Prevention Potential and Employing It as a Powerful Biocontrol Agent Against *Fusarium oxysporum*

**DOI:** 10.3390/ijms26020700

**Published:** 2025-01-15

**Authors:** Qi Wang, Zhenshu Sun, Tiantian Li, Tiantian Fan, Ziqi Zhou, Jiayin Liu, Xiuling Chen, Aoxue Wang

**Affiliations:** 1 College of Horticulture and Landscape Architecture, Northeast Agricultural University, Harbin 150030, China; kakalpooh@163.com (Q.W.); litiantian080821@163.com (T.L.); 15146476624@163.com (T.F.); 2 College of Life Sciences, Northeast Agricultural University, Harbin 150030, China; shushu990619@163.com (Z.S.); 17363390191@163.com (Z.Z.); 3 College of Arts and Sciences, Northeast Agricultural University, Harbin 150030, China; liujiayin@neau.edu.cn

**Keywords:** biocontrol bacterium, *F. oxysporum*, *Bacillus velezensis*, bioagent

## Abstract

Biocontrol microbes are environment friendly and safe for humans and animals. To seek biocontrol microbes effective in suppressing *Fusarium oxysporum* is important for tomato production. *F. oxysporum* is a soil-borne pathogen capable of causing wilt in numerous plant species. Therefore, we found a biocontrol bacterium with an excellent control effect from the rhizosphere soil of plant roots. In this work, we focus on two parts of work. The first part is the identification and genomic analysis of the biocontrol bacterium Y-4; the second part is the control efficiency of strain Y-4 on *F. oxysporum*. For this study, we identified strain Y-4 as *Bacillus velezensis*. It is an aerobic Gram-positive bacterium that can secrete a variety of extracellular enzymes and siderophores. Strain Y-4 also contains a large number of disease-resistant genes and a gene cluster that forms antibacterial substances. In addition, we found that it significantly inhibited the reproduction of *F. oxysporum* in a culture dish. In the indoor control effect test, after treatment with strain Y-4 suspension, the disease index of tomato plants decreased significantly. Furthermore, the control efficiency of the plants was 71.88%. At the same time, Y-4 bacterial suspension induced an increase in POD and SOD enzyme activities in tomato leaves, resulting in increased plant resistance. Taken together, strain Y-4 proves to be an effective means of controlling *F. oxysporum* in tomatoes.

## 1. Introduction

The tomato (*Solanum lycopersicum*) of the *Solanaceae* family is cultivated worldwide [1]. In recent years, tomato has become the leading crop in the world, providing farmers with an important economic resource. However, tomato production is seriously restricted by soil-borne pathogens such as *F. oxysporum*, which is known to infect different types of hosts and cause serious damage to crops such as cotton, tomatoes, and bananas [2]. *F. oxysporum* enters the plant through the root and propagates within the vascular bundle, disrupting the ability of the root tissue to transport nutrients to the vascular bundle, resulting in the host’s eventual death, seriously reducing fruit yield and quality. Moreover, the frequent and excessive use of chemical fungicides produces serious environmental and food safety problems [3,4]. Therefore, a study on several topics related to the control of *F. oxysporum* is necessary.

Biological control is a safe and environmentally friendly option for preventing and treating tomato plant wilt disease. It offers a new alternative that is beneficial for humans and the environment [5]. Biological control has been studied for over 100 years and is considered a viable alternative method to chemical control [6]. It is one of the best potential strategies for the control of soil-borne diseases [7]. Bacteria as the primary source of biological antibacterial agents have the advantages of rapid reproduction and easy preparation of fermentation solutions and the most noticeable control effect [8]. Li et al. found that the A57 strain of *Bacillus subtilis* could considerably suppress cotton standing blight and yellow wilt, inhibiting the germination of conidia and causing spore deformation to achieve a control effect [9]. Other studies showed that *Bacillus megaterium* BM1 and *B. velezensis* RC218 were both capable of considerably reducing the occurrence of wheat blast disease [10,11]. Lu et al. reported that a new species of *streptomyces*, *Streptomyces rimosus* M527, not only displayed broad-spectrum antifungal activity but also showed the strongest antagonistic activity against the spore germination of *F. oxysporum* f.sp. *cucumerinum* [12]. In addition, biological control has not only focused on the control effects of strains. Studies showed that *Bacillus* secretes antagonistic compounds. Wang et al. identified three fungal lipopeptides (iturin, surfactin, and bacillomycin D) secreted by *Bacillus amylolyticus* W19 [13].

In recent years, chemical residues on fruits that are difficult to clean and harmful to consumers and the massive use of chemical agents to control fungal diseases has caused a decline in the structure of soil aggregate and the destruction of microbial communities [14]. Although there are many reports on the biocontrol of *B. velezensis* against plant fungal disease, there are very few *B. velezensis* agents now used in agricultural practices. Therefore, it is necessary to screen out more effective *B. velezensis* biocontrol strains and understand the influences on plants and pathogens.

In this work, we isolated and identified a biocontrol bacterial strain, Y-4, and used it as a research object. The research was mainly carried out in the following ways: (a) an analysis of a potential antibacterial mechanism through whole genome sequencing and metabolic gene clusters, (b) determining the antagonism activity of strain Y-4 on *F. oxysporum* through a culture dish, (c) evaluating the control efficiency of the Y-4 strain on wild tomatoes indoors, and (d) determining the induction of antioxidant enzymes in tomato leaves by strain Y-4. This study aimed to provide the resources and methods for the control of *F. oxysporum*, using strain Y-4 as a biological agent.

## 2. Results

### 2.1. Isolation of Biocontrol Bacterium Y-4

A total of six biocontrol strains that could inhibit the reproduction of *F. oxysporum* were selected from the rhizosphere soil. Thus, we took strain Y-4 as the main research object (Table 1).

### 2.2. Identification of Biocontrol Bacterium Y-4

**Figure 1:**
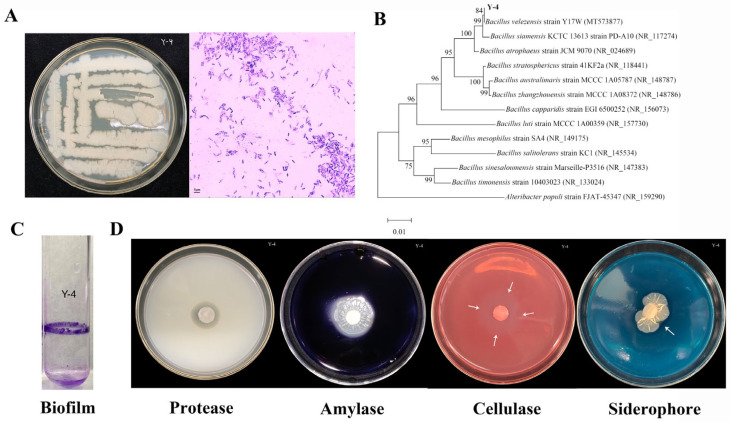
Identification and physiological and biochemical characteristics of strainY-4. (**A**) Observation of the apparent morphology and Gram staining of strain Y-4. (**B**) Strain Y-4 phylogenetic tree construction. (**C**) Strain Y-4 biofilm determination map. (**D**) From left to right, protease, amylase, cellulase, and siderophore secretions (The arrow represents the indicator circle of cellulase and siderophore secreted by the strain).

Y-4 colonies were creamy white, oily, paste-like, smooth at the edges, and slightly folded at the center and smooth and opaque on the surface (Figure 1A). After microscopic observation and after Gram staining, strain Y-4 was Gram-positive with a purple body and rod-shaped bacteria (Figure 1A).

#### 2.2.1. Molecular Identification of Y-4

The 16S rDNA sequence of strain Y-4 was submitted to NCBI’s GenBank database for homology comparison with reported sequences. The strain with more similar homology to strain Y-4 is *B. velezensis* Y17W strain (MT573877) (Figure 1B).

To further determine the taxonomic information of strain Y-4, we compared each sequence of strain Y-4 with the NT database. The results are shown in Table 2. According to the identity and score comparison results, the complete genomes of *B. velezensis* strain BA-26 chromosome and *B. velezensis* strain CGMCC 11640 chromosome are more similar to that of the Y-4 genome (Table 2). Combined with the comparison results of the 16S rRNA and NT databases, strain Y-4 was determined to be a strain of *B. velezensis*.

#### 2.2.2. Physiological and Biochemical Identification of Y-4

The results showed that strain Y-4 was an aerobic and alkali-producing type of bacteria. The catalase test was positive. Methyl red was negative (Table 3). Strain Y-4 can form biofilms that protect plants from infections (Figure 1C). Furthermore, it can secrete protease, amylase, cellulase, and siderophores (Table 3 and Figure 1D).

### 2.3. Genome-Wide Information of the Strain

**Figure 2:**
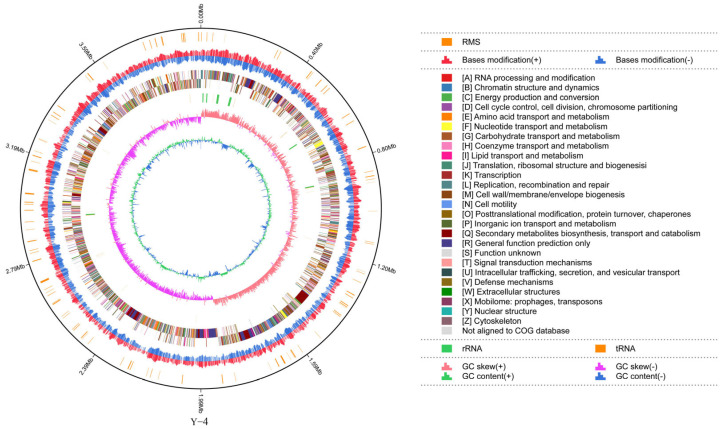
Genome circle map can display the genome information of the strain Y-4 sample in all directions.

From the assembly results, we learned that the subread length of strain Y-4 was 3,984,866 bp, G + C content was 46.45%, and the proportion of the A, T, G, and C base content was 26.75%, 26.80%, 23.25%, and 23.20%, respectively (Figure 2 and Supplemental Table S1). We submitted the sequence data to the NCBI database and obtained the accession number CP139053.

### 2.4. Genome Structure Annotation Information of the Strain

In total, 4022 coding genes were predicted, with a 3,555,231 bp length for all of the projected coding genes. The predicted number of tRNA was 87, with a total length of 6694 bp. There were 3 types of rRNA, 27 were predicted, and the total length was 41,230 bp (Table 4).

### 2.5. Gene Function Annotation of the Strain

#### 2.5.1. Annotations on Basic Functions of Strain

**Figure 3:**
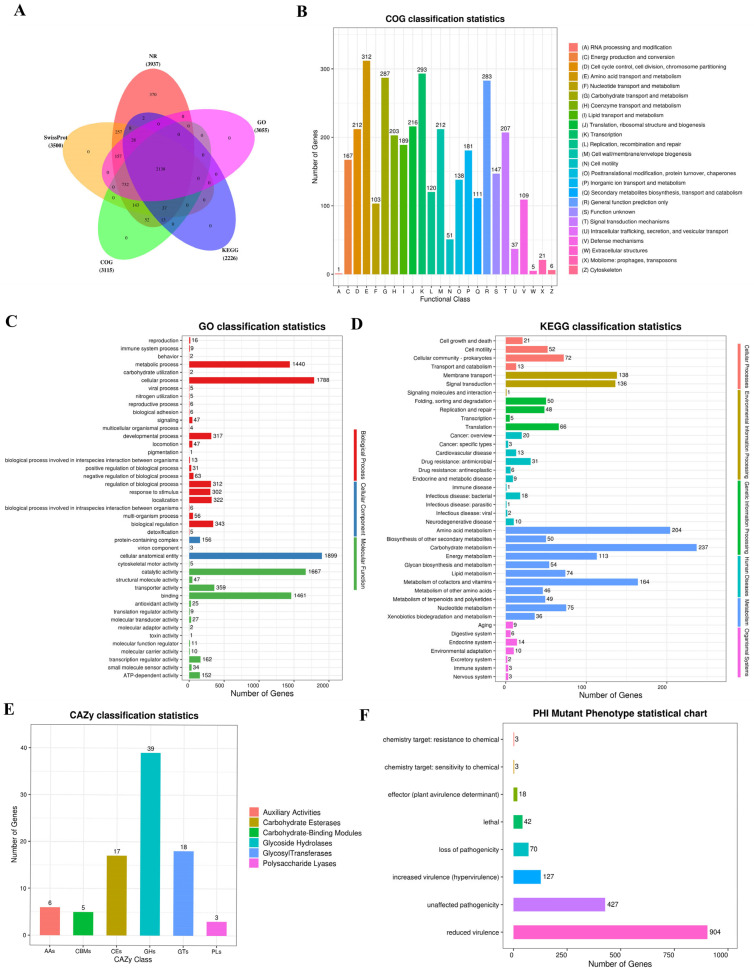
Basic function database and special function database annotation results illustrated in a diagram. (**A**) Statistical charts of basic database data. (**B**) COG function classification diagram. (**C**) GO Level 2 classification level annotation distribution map. (**D**) Statistical chart of the Level 2 KEGG annotation result classification. (**E**) CAZy annotated classification statistical chart. (**F**) Phenotypic statistics of the PHI mutation.

To study Y-4’s genome function, its sequence was annotated in five databases: NR, SwissProt, COG/KOG, KEGG, and GO. There were 3937 genes annotated in the NR database, 3500 in SwissProt, 3115 in COG/KOG, 2226 in KEGG, and 3055 in GO (Figure 3A).

The COG database is a system annotated by NCBI based on the distance of the gene linear homology. Through sequence similarity alignment, a protein sequence is assigned to a COG cluster. The Y-4 biocontrol bacterium had 3115 (77.84%) annotated genes that were classified into 24 categories (Figure 3B). Among all categories, the number of genes involved in amino acid transport and metabolism was the largest, involving 312 genes (Figure 3B).

A term is the fundamental unit of GO. In the GO database, 3055 genes (76.34%) of the Y-4 genes were annotated (Figure 3C). Cellular anatomical entity had the highest number of genes (1899) in the secondary classification. Then, cellular and metabolic process annotated 1788 and 1440 genes. Catalytic and binding activities annotated 1667 and 1461 genes, respectively (Figure 3C).

In the KEGG database, 2226 (55.62%) of the Y-4 genes were annotated. The results revealed that the metabolism portion had the most annotated genes, with 1102 genes, followed by the environmental information processing section. In the secondary categorization, 237 genes were annotated to carbohydrate metabolism and 204 genes to amino acid metabolism. The number of genes in these two categories is relatively higher than in the others (Figure 3D).

#### 2.5.2. Annotation on Special Functions of Strain

In the CAZy database, strain Y-4 annotated to 85 genes, with glycoside hydrolases having the most genes annotated (39 genes), followed by glycosyl transferases (18 genes) and carbohydrate esterases (17 genes) (Figure 3E). Polysaccharide lyases had the fewest genes annotated, with only three (Figure 3E).

In the PHI mutant phenotype statistics, we observed the most prominent number of reduced virulence genes in the Y-4 sample with a gene count of 904 (Figure 3F). This is substantially greater than the phenotypes of other mutations. It should be noted that reduced virulence is regarded as a key mutation trait for reducing the pathogenicity of pathogenic bacteria (Figure 3F).

### 2.6. Gene Cluster Analysis of Secondary Metabolites of Strain

According to the anticipated results, the strain Y-4 genome had 13 metabolite synthesis gene clusters. Surfactin (lipopeptide), butirosin A/butirosin B (aminoglycoside), macrolactin H (polyketone), bacillaene (polyketone), fengycin (lipopeptide), difficidin (polyketone), bacillibactin (siderophore), and bacilysin (dipeptide) were identified as fungistatic substances (Table 5). Six compounds (macrolactin H, bacillaene, fengycin, difficidin, bacillibactin, and bacilysin) were 100% similar to known gene clusters, and one compound (surfactin) had 91% similarity. Moreover, the four gene clusters responsible for secondary metabolite synthesis showed no resemblance to the known clusters, and they biosynthesized two terpenes, one T3PKS and one NRPS, respectively (Table 5).

### 2.7. Antifungal Activities of Biocontrol Bacterium Y-4

#### 2.7.1. Determination of Broad-Spectrum Bacterial Inhibition of Biocontrol Bacterium Y-4

**Figure 4:**
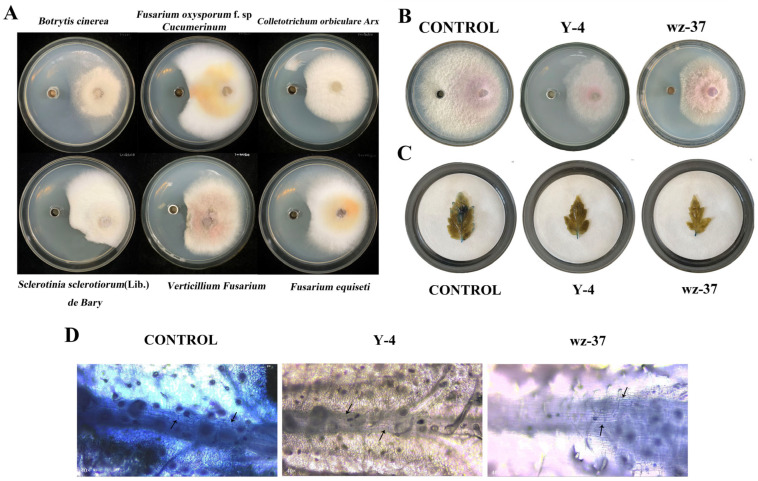
Inhibitory effect of Y-4 on *F. oxysporum*. (**A**) Y-4 broad-spectrum fungistatic map. (**B**) The effect of culture dish confrontation between Y-4 and *F. oxysporum*. (**C**) Observation of the apparent morphology of Trypan Blue staining. (**D**) Microscopic morphology of leaf veins stained with Trypan Blue. Arrows indicate staining of vein cells.

In this experiment, the Y-4 bacterial suspension showed different degrees of bactericidal effects against six pathogenic bacteria (*B. cinerea*, *F. oxysporum* f.sp. *cucumerinum*, *Colletotrichum orbiculare Arx*, *Sclerotinia sclerotiorum* (Lib.) *de Bary*, *Verticillium fusarium and Fusarium equiseti*). These results showed that Y-4 has broad-spectrum bacterial inhibition (Table 6 and Figure 4A).

#### 2.7.2. Determination of the Inhibition Effect of *F. oxysporum*

Through the culture dish confrontation results, strain Y-4 had the strongest inhibitory effect on *F. oxysporum*, which was significantly different from CONTROL. In addition, strain wz-37, which was used as a positive control, inhibited the *F. oxysporum* to a lesser extent than strain Y-4 (Figure 4B and Table 7).

#### 2.7.3. Trypan Blue Dyeing Observation of Y-4

The blue area of the tomato leaves treated with the bacterial suspension was much smaller compared to the control group, among which the stained area of leaves treated with the strain Y-4 bacterial suspension was the smallest, indicating that the leaves had the largest number of viable cells. In contrast, the leaves in the CONTROL were completely stained and the leaf cells were completely inactive. After treatment with strain wz-37, the leaf cells were noticeably stained. However, the prevention and control effect of strain Y-4 was found to be higher than that of strain wz-37 (Figure 4C,D).

### 2.8. Determination of Indoor Control Efficiency of Biocontrol Bacterium Y-4

**Figure 5:**
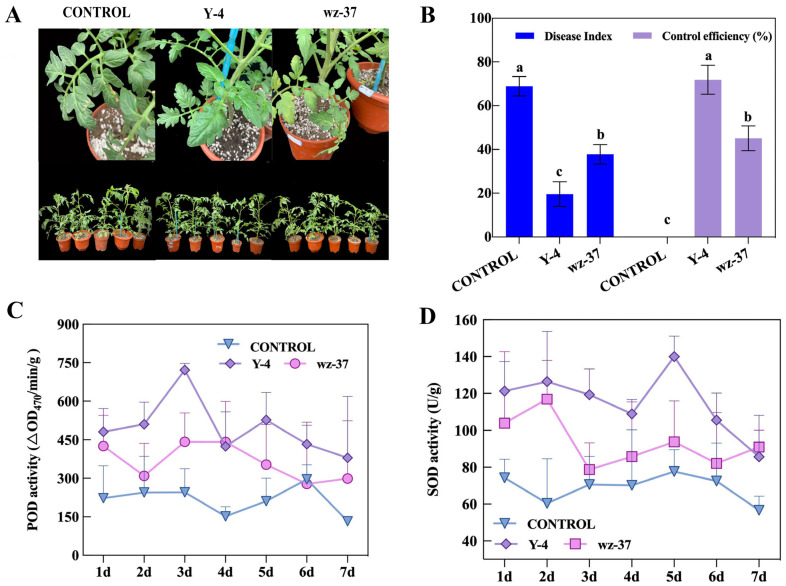
The control effect of Y-4 on *F. oxysporum* in an indoor control efficiency test and the change in defense enzyme activity within 7 days. (**A**) Phenotypic observation of the indoor control efficiency test. (**B**) Changes in disease index and control index of biocontrol bacterium against *F. oxysporum*. Different letters indicate significant differences at *p* ≤ 0.05. (**C**) POD enzyme activity. (**D**) SOD enzyme activity.

In greenhouse prevention studies, the disease index of the plants treated with strain Y-4 was the lowest and the control efficiency was the best, which were 19.56 and 71.88%, respectively (Figure 5A,B). All the plants with the addition of the biological control bacteria had a lower disease index than the CONTROL group. The control efficiency of wz-37 was considerably lower than that of Y-4 (Figure 5B).

### 2.9. Induction of Antioxidant Enzymes in Tomato Leaves by Biocontrol Bacterium Y-4

POD enzyme activity in tomato leaves treated with bacterial suspension of strains Y-4 and wz-37, both reaching a maximum on day 3, with 721.00 min/g for Y-4 and 442.33 min/g for wz-37. The highest POD activity was observed in the strain Y-4 treatment (Figure 5C). Therefore, spraying the leaves with the Y-4 biocontrol suspension could considerably increase the POD enzyme activity and further increase the plant’s resistance to pathogenic bacteria.

The trend of SOD and POD activities in tomato leaves was similar. Strain wz-37 treatments reached the highest value on the second day, which was 116.89 U/g. The difference was that the treatment of strain Y-4 reached its highest value on the fifth day, which was 136.58 U/g. Moreover, strain Y-4 had the highest SOD activity among all groups (Figure 5D). In general, Y-4 bacterial suspensions can boost POD and SOD activities in leaves.

## 3. Discussion

Among vegetable crops, the tomato stands out as one of the most widely consumed and cultivated worldwide. Tomato fruit production is significant globally, with an annual production of 182 million tons according to the FAO (Food and Agriculture Organization of the United Nations). Tomatoes are easily infected by diseases during growth, resulting in a decline in fruit quality. *F. oxysporum* is a key pathogen that causes *Fusarium* wilt in tomato plants [15]. To reduce the threat of this pathogen, an efficient and ecofriendly control agent for *F. oxysporum* is required. Furthermore, we were discovered that Y-4 can be used to prevent a range of fungal illnesses and had potent broad-spectrum antibacterial action (Figure 4A).

*Bacillus* is a widely employed biological resource for crop protection due to its antagonistic and growth-promoting properties. *Bacillus* is a commonly used biological resource for protecting crops [16]. *B. velezensis* is a branch of *Bacillus* and is a relatively lately discovered type. The prospects for development are strong, and it has many ways it can be used [17,18]. It was previously found that *B. velezensis* can prevent and control various kinds of plant-disease-causing pathogens, such as *Verticillium dahliae* Kleb, *Alternaria brassicae*, *Pyricularia grisea* (Cooke) Sacc, and Lettuce Root Rot (*Pythium*) [19,20,21]. In this work, we isolated and identified a strain of *B. velezensis* Y-4 from soil that showed strong antifungal activity. In this test, strain Y-4 could effectively inhibit *F. oxysporum* infections in plants, thereby reducing the disease index of tomato plants. Through indoor prevention experiments, we found that strain Y-4 was successful in preventing disease caused by *F. oxysporum*, with it controlling up to 71.88%, considerably higher than that of the CONTROL, and decreasing the disease index (Figure 5B). Previous studies showed that the control effect of *B. velezensis* D18 bacterial suspension on banana blight was 67.9%, which could effectively control the occurrence of fungal diseases [22]. In this study, the control efficiency of the Y-4 bacterial suspension was better than that of the D18 bacterial suspension. Wang et al. isolated a bacterial strain (SF18-3) and identified it as *B. velezensis*. Its fermentation liquor effectively controlled *Xanthomonas campestris* pv.vesicatoria (Doidge) Dye (Bacterial Spot Disease of Pepper), with a control efficiency of 70.17% [23]. 

POD and SOD are two defense enzymes that exist widely in plants. When plants are biologically stressed, they frequently enhance the activity of defensive enzymes to resist fungal infection. In our study, POD and SOD activities increased at first and then decreased gradually (Figure 5C,D). POD activity was consistent with the research results of Wang et al. [24]. Our results showed that, after spraying foliar with a biocontrol agent, the strains first propagated and occupied a favorable living space to inhibit the infection of *F. oxysporum*. When *F. oxysporum* invades plants, it will cause a large amount of ROS to accumulate. ROS will destroy biofilms and cause membrane peroxidation. The SOD enzyme plays an important role in clearing ROS and alleviating the damage caused by ROS to plants.

More kinds of bioactive metabolites are produced by *B. velezensis* than by other bacterial strains [25]. We analyzed 13 gene clusters in strain Y-4, of which 9 gene clusters can synthesize known antibiotics, including locillomycin/locillomycin B/locillomycin C, surfactin, butirosin A/butirosin B, macrolactin H, bacillaene, fengycin, and others (Table 5). Fengycin, bacillibactin, and surfactin are classic lipopeptide antibacterial substances. Thurlow et al. reported that a strain of *B. velezensis* AP193 can produce the polyketide compound difficidin [26]. *B. velezensis* FZB4 can produce related surfactin and fengycin substances, which achieve antibacterial effects by changing the cell wall of the mycelium or conidia of specific fungi [27]. Fengycin has been reported to be the main antibacterial substance produced by *B. velezensis*. It can significantly inhibit the growth of *Ralstonia solanacearum* and *F. oxysporum* [28] and inhibit the growth of *Fusarium solani* by interfering with the cell membrane [29]. Bacilysin is a non-ribosomal formed dipeptide antibiotic composed of L-alanine and L-anticapsin, produced by *Bacillus* [30]. Metabolites produced by microorganisms can regulate the exchange of substances and competition and interaction between organisms [31]; they are also used as a starter to induce resistance in plants. Therefore, *B. velezensis* has broad application prospects in biopesticides.

According to earlier research, *B. velezensis* has functional genes that prevent harmful bacteria. For example, a strain of *B. velezensis* isolated from rice rhizosphere can induce resistance to disease in *Arabidopsis thaliana* and an overproduction of the PAD4 gene in plants [32]. In the CAZy database, strain Y-4 involves 39 genes annotated in glycoside hydrolases (Figure 3E). The majority of glycoside hydrolases are linked to cell wall metabolism, defense mechanisms, secondary metabolism, glycolipid metabolism, and signal transduction [33]. In the PHI database, we found that up to 904 genes in strain Y-4 were annotated into reduced virulence (Figure 3F). Therefore, we speculated that the antagonistic effect of strain Y-4 was due to the existence of a disease-resistant gene. The analysis results of the PHI data showed us the direction for further in-depth research.

## 4. Materials and Methods

### 4.1. Bacteria, Pathogen, and Culture Conditions

*B. velezensis* wz-37 was previously isolated in the laboratory from rhizosphere soil samples of tomatoes, cucumbers, corn, and wheat. The wz-37 was deposited in the China General Microbiological Culture Collection Centre (CGMCC No. 15766) (Beijing, China). The bacterial isolates were grown on LB agar media [34] at 28 °C in the dark (the same as below unless stated otherwise) for 3 days before being used.

*F. oxysporum*, *B. cinerea*, *F. oxysporum* f.sp. *cucumerinum*, *Colletotrichum orbiculare Arx*, *Verticillium fusarium*, *Fusarium equiseti*, and *Sclerotinia sclerotiorum* (Lib.) *de Bary* were preserved in the Horticultural Biotechnology Laboratory of Northeast Agricultural University, China (Harbin, China). The pathogen was grown on potato dextrose agar (PDA) [35] at 28 °C in the dark (the same as below unless stated otherwise) for 7 d before being used.

Bacterial suspension: On a clean bench, the purified cultures Y-4 and wz-37 (Quantity: 1%) were inoculated into liquid LB agar media and placed in a shaker at 200 rpm, and the bacterial suspension was obtained by shaking the culture for 48 h. We used sterile water to adjust the bacterial suspension concentration to 1 × 10^9^ CFU/mL.

*F. oxysporum* suspension: *F. oxysporum* was incubated in culture dishes for 7 days, and the mycelium was allowed to grow all over the Petri dish, with germinating spores attached to the surface. The mycelium was rinsed with sterile water to disperse the spores in the solution and filtered, and the spore solution was adjusted using a spectrophotometer. We used sterile water to adjust the *F. oxysporum* suspension concentration to 1 × 10^6^ CFU/mL.

### 4.2. Tomato Seeds

The tomato variety used in the experiment was “DRK0568” (also known as the Provence tomato). The seedlings were cultured in the Horticultural Biotechnology Laboratory of Northeast Agricultural University, China (Harbin, China).

### 4.3. Isolation of Biocontrol Bacterium

Soil samples were collected from the rhizosphere soil of healthy *Schefflera heptaphylla* plants in Xishuangbanna Autonomous Prefecture, Yunnan Province, China. The surface soil was removed, and soil samples were collected at a depth of 5–10 cm. Sampling was performed using the multipoint sampling method, and samples were kept in self-sealing bags and numbered.

Soil samples were used to isolate the biocontrol bacterium. The isolation method described for biocontrol strains followed the procedure of Li et al. [36]. After the colony grew out on the culture dish, it was randomly selected for purification. Using *F. oxysporum* as an indicator pathogen, the bacteria with a good antagonistic effect were screened using the culture dish antagonistic method.

### 4.4. Identification of Biocontrol Bacterium

The biocontrol bacterium was cultured on an LB agar media and observed for morphology (the underlining method is shown in Supplemental Figure S1).

#### 4.4.1. Molecular Identification

The DNA extraction and identification of the strain via 16S rRNA were conducted by Beijing LiuheHuada Gene Technology Co., China (Beijing, China). The DNA extraction method was according to the instructions of the Bacterial Genome Kit (Tiangen Biochemical Technology Co., Ltd., Beijing, China). The 16S rRNA sequences of strain Y-4 were submitted to the GenBank database of the NCBI and subjected to BLAST comparison and homology with the reported sequences. The phylogenetic tree of Y-4 evaluated in 1000 replicates was constructed using the neighbor-joining method in the MEGA 10.2.6 software.

The whole genome sequencing analysis of strain Y-4 was operated by the Wuhan Frasergen company, China (Wuhan, China). For the bacterial genome assembly part, pb_assembly_hifi_microbial (from smrtlink11.0.0) software was used for assembly, and the second-generation data pair reads-based assembly correction was used to obtain the final assembly results using pilon-1.24 software [37]. Each sequence was compared with the NT database using the BLASTn tool of the ncbi-blast-2.11.0+ software [38] with the comparison options “-task megablast -outfmt 5 -evalue 1e-5 -max_target_seqs 3”. The remaining settings were set to default values. The strain identification was determined by combining the results of the NT database comparison and 16S rRNA gene sequencing.

#### 4.4.2. Physiological and Biochemical Identification

Aerobic and anaerobic, catalase, glucose oxidative fermentation, and methyl red tests all refer to Berger’s Manual for the identification of bacteria [39]. Determination of the biofilm-forming ability followed the method of Sun et al. [40]. We referred to Tagele et al. for the determination of protease [41] and Slama et al. for the determination of amylase [42]. In the determination of cellulase, the cellulase detection culture dish was configured. A 6 mm diameter sterile filter paper was placed at the center of the test culture dish and 2 μL of Y-4 bacteria suspension cultured for 48 h was dripped onto the filter paper. The detection culture dish was placed in a constant-temperature incubator at 28 °C to culture for 3–7 days. If there was a transparent halo around the filter paper, it indicated that the strain could produce cellulase. The determination of siderophores followed the procedure of Bernhard Schwyn and J.B. Neilands (1987) [43]. The presence of an orange halo around the colony indicated siderophore production. All experiments in this part were repeated three times.

### 4.5. Annotation of the Structure and Function of Bacterial Genomes

#### 4.5.1. Genome Structure Annotation

We used Glimmer (v3.02) [44] to find the coding genes of microbial DNA (particularly bacteria, archaea, and viruses), tRNAscan-SE (2.0.9) [45] to predict the tRNA of the microbial genome, and RNAmmer (v1.2) [46] to predict rRNA.

#### 4.5.2. Annotation on the Database of Basic Gene Functions

Regarding the basic function annotation, we compared the predicted gene protein sequences to the Cluster of Orthologous Groups of Proteins (COG), KEGG, and GO databases.

We used diamond (v2.0.9.147) software to compare the protein sequence of the predicted gene to the COG2020 database using the BLASTp command. The E value 1e-5, and the hit with the highest score is selected as the final annotation result. For KEGG, we used the BLASTp command of the diamond (v2.0.9.147) software to align the protein sequence of the predicted gene into the entire library of KOBAS-v3.0 [47] and then used KOBAS-v3.0 software to analyze the comparison results to map the gene ID to KO. Finally, the hierarchical relationship table in the KEGG BRITE library (last updated: 11 October 2021) was used to annotate each level. For GO, we mapped GO term annotations through ID mapping (20210616) using SwissProt annotation results and then annotated at various levels using go-basic.obo (v2021-09-01).

#### 4.5.3. Annotation on the Database of Special Gene Functions

To annotate CAZy [48], we employed dbCAN2 (an automatic web annotation tool for carbohydrate-related enzymes, annotation software HMMER-v3.3.2, parameter E Value <= 1e-5, coverage >= 0.35). In addition, we utilized diamond (v2.0.9.147) software’s BLASTp command to compare the predicted gene’s protein sequence to the PHI database.

### 4.6. Annotation of the Secondary Metabolite Gene Cluster

In this study, the gene clusters produced by the secondary metabolites of the strain were analyzed using anti-SMASH (v5.1.1).

### 4.7. Antifungal Activities of Biocontrol Bacterium

#### 4.7.1. Determination of Broad-Spectrum Antifungal Effect of Biocontrol Bacterium

The abovementioned pathogens were made into 6 mm fungal chunks (we punched holes vertically on the surface of the fungal culture medium and used a sterile inoculation needle to pick it out to obtain a fungal chunk). The fungal chunk was inoculated on the right side of the sterile PDA agar media center, and the sterile Oxford cup was placed on the left. Using a pipette, 80–100 μL of Y-4 and wz-37 bacterial suspensions were injected into the Oxford cups. On the right side of the control group was an *F. oxysporum* chunk, and 80–100 μL of sterile water was added to the Oxford cup on the left side. We used wz-37 as a positive control group. The culture dish was sealed and incubated in a constant-temperature oven at 28 °C for 7 days. The width of the inhibition zone was measured. The test was repeated three times.

#### 4.7.2. Determination of the Inhibition Effect of *F. oxysporum*

First, we made *F. oxysporum* chunks (the production method of *F. oxysporum* chunks is the same as in Section 4.7.1). The culture dishes were filled with PDA agar media and dried. We placed the *F. oxysporum* chunks on the right side of the Petri dish and placed the sterile Oxford cup on the left side. Using a pipette, 80–100 μL of Y-4 and wz-37 bacterial suspensions were injected into Oxford cups, and CONTROL added an equal amount of sterile water to them. The culture dish was sealed and incubated in a constant-temperature oven at 28 °C for 7 days. The width of the inhibition zone was measured. The test was repeated three times.

#### 4.7.3. Trypan Blue Dyeing Observation

The pretreatment for the leaves was as follows: The leaves were immersed in 75% ethanol for 30 s. After rinsing, the leaves were immersed in 3% sodium hypochlorite for 3 min and then rinsed three times with sterile water. The petioles were wrapped in sterile, moist, skimmed cotton wool. We applied sterile water (CONTROL), Y-4 bacterial suspension, and wz-37 bacterial suspension (1 × 10^9^ CFU/mL) to the leaves. A suspension of *F. oxysporum* spores (1 × 10^6^ CFU/mL) was evenly sprayed on dried leaves. A staining test was performed after 3 days. The dyeing process followed the method described by Yin et al. [49]. The stained leaves were observed for cell color under an electron microscope. Deep cell color indicated leaf cell death, and light color indicated leaf cells were active.

### 4.8. Determination of Indoor Control Efficiency of Biocontrol Bacterium

Biocontrol suspension and *F. oxysporum* suspension (preparation method followed Section 4.1) were placed into two spray bottles, respectively. First, we sprayed biocontrol suspension evenly on the surface of the plant. After 24 h, we sprayed *F. oxysporum* suspension.

After 15 days of treatment, plant disease indices and prevention effects were recorded. The test was repeated five times. The disease index and disease control effect were calculated using the following formulas [50]. The treatment was as follows:

(1) CONTROL+ *F. oxysporum*: we sprayed the surface of tomato plants with sterile water first, followed by the *F. oxysporum* suspension.
(2) Y-4 + *F. oxysporum*: we sprayed the surface of tomato plants with Y-4 suspension first, followed by the *F. oxysporum* suspension.
(3) wz-37 + *F. oxysporum*: we sprayed the surface of tomato plants with wz-37 suspension first, followed by the *F. oxysporum* suspension.

Disease index = [∑(The number of diseased plants in this grade × Disease grade)/(Total number of plants investigated × the highest disease grade)] × 100.

Control efficiency (%) = [(Disease index of control − Disease index of treated group)/Disease index of control] × 100.

### 4.9. Induction of Antioxidant Enzymes in Tomato Leaves by Biocontrol Bacterium

Leaf samples were collected after 7 days of treatment of the plants, as in Section 4.8. The collection period lasted 7 days. For the sampling standard, leaves with the same growth position and similar leaf size were selected and liquid nitrogen was used for quick freezing, followed by storage in −80 °C refrigerators. Peroxidase (POD) and superoxide (SOD) kits (Suzhou Grace Biotechnology Co., Ltd., Suzhou, China) were used to determine the activity of defense enzymes.

### 4.10. Statistical Analysis

Excel: numerical values were the mean ± standard deviation (SD) of triplicates (Version 16.77.1). Significance analysis was performed using the one-way ANOVA test (*p* ≤ 0.05); multiple group comparisons were performed using Tukey after ANOVA in SPSS (Version 26.0). The error bars indicate the SD of the data. The column and line chart were drawn using GraphPad Prism 9 (Version 9.0.0).

## 5. Conclusions

In conclusion, strain Y-4 as a biocontrol bacterium has been widely used in plant disease management owing to its high efficiency, safety, and environmental friendliness. Strain Y-4 is an aerobic bacterium, which can produce biofilm and many extracellular enzymes. Strain Y-4 plays a crucial role in reducing *F. oxysporum*-induced blight, safeguarding tomato plants against harmful bacteria and ensuring high-quality and high-yield tomato fruits. In summary, strain Y-4 is a newly discovered and highly effective agent for pathogen control.

## Figures and Tables

**Table 1:** Preliminary screening of the width of the fungistatic zone.

Strain Number	Inhibition Zone/cm
Y-1	0.41 ± 0.05 c
Y-2	0.47 ± 0.03 bc
Y-3	0.73 ± 0.10 ab
Y-4	0.79 ± 0.09 a
Y-5	0.57 ± 0.14 abc
Y-9	0.49 ± 0.01 bc

Note: 1. Numerical values were mean ± SD of triplicates (the same as below). 2. One-way ANOVA test (*p* ≤ 0.05) was used to evaluate the significance between data. Statistical methods: multiple group comparisons using Tukey after ANOVA (the same as below). 3. Different letters indicate significant differences at *p* ≤ 0.05 (The same as below).

**Table 2:** Results of NT library comparison of assembled genomes.

Query_id	Y_4_chr	Y_4_chr	Y_4_chr
Query_length	3,984,866	3,984,866	3,984,866
Subject_id	CP046918.1	CP026610.1	CP071970.1
Subject_length	4,035,062	4,322,979	4,160,003
Identity	1,278,306/1,281,807 (99.73)	902,961/904,588 (99.82)	733,597/734,823 (99.83)
Score	2,347,330	1,661,360	1,350,150
E value	0	0	0
Description	*B. velezensis* strain BA-26 chromosome, complete genome	*B. velezensis* strain CGMCC 11640 chromosome, complete genome	*B. amyloliquefaciens* strain XJ5 chromosome, complete genome

**Table 3:** Physiological and biochemical identification.

Physiological and Biochemical Identification	Y-4
Aerobic or anaerobic	Aerobic type
Catalase test	+
Glucose oxidative fermentation	Alkali-producing type
Methyl red	−
Biofilm	+
Protease	+
Amylase	+
Cellulase	+
Siderophore	+

Note: In Table 3, “+” represents positive, “−” represents negative. The above experiment was repeated three times.

**Table 4:** Statistical table for the prediction of basic genetic elements.

Basic Gene Element Prediction				
Coding Gene	Genome_Size (bp)	Gene_Num (#)	Gene_TotalLen (bp)	Gene_AverageLen (bp)
	3,984,866	4002	3,555,231	888.36
tRNA	Number	Total_len (bp)	Average_Len (bp)	%of_genome
	87	6694	76.94	0.17
rRNA	Number	Total_len (bp)	Average_Len (bp)	%of_genome
	27	41,230	1527.04	1.03

**Table 5:** Prediction of secondary metabolite gene clusters of strain Y-4.

Region	Type	From	To	Most Similar Known Cluster	Similarity
Region 1	NRPS, transAT-PKS	197,788	275,401	Locillomycin/locillomycin B/locillomycin C	28%
Region 2	NRPS	353,992	418,853	Surfactin	91%
Region 3	PKS-like	942,114	983,358	butirosin A/butirosin B	7%
Region 4	Terpene	1,068,980	1,086,136	-	
Region 5	transAT-PKS	1,404,718	1,491,156	macrolactin H	100%
Region 6	transAT-PKS, T3PKS, NRPS	1,718,562	1,818,285	Bacillaene	100%
Region 7	NRPS, transAT-PKS, betalactone	1,898,242	2,034,192	Fengycin	100%
Region 8	Terpene	2,085,270	2,080,153	-	
Region 9	T3PKS	2,145,247	2,186,347	-	
Region 10	transAT-PKS	2,314,539	2,408,331	Difficidin	100%
Region 11	NRP-metallophore, NPRS, RiPP-like	3,031,566	3,083,360	Bacillibactin	100%
Region 12	NPRS	3,376,282	3,444,726	-	
Region 13	Other	3,649,950	3,691,368	Bacilysin	100%

-: None.

**Table 6:** Broad-spectrum fungistatic band distance of Y-4 (cm).

Name of Pathogen	Inhibition Zone Width/cm
*B. cinerea*	0.90 ± 0.01 a
*F. oxysporum* f.sp *cucumerinum*	0.60 ± 0.02 c
C. orbiculare *Arx*	0.61 ± 0.18 c
*V. fusarium*	0.72 ± 0.0025 ab
*F. equiseti*	0.87 ± 0.07 b
S. sclerotiorum (Lib.) *de Bary*	0.71 ± 0.005 ab

**Table 7:** Fungistatic distance of Y-4 to *F. oxysporum* (cm).

Strain Number	Inhibition Zone Width/cm
CONTROL	0.00 ± 0.00 c
Y-4	0.69 ± 0.04 a
wz-37	0.45 ± 0.05 b

## Data Availability

Data will be made available on request.

## Supplementary Materials

The following supporting information can be downloaded at https://www.mdpi.com/article/10.3390/ijms26020700/s1.
