# Supplementary material for: Identifying a Biocontrol Bacterium with Disease-Prevention Potential and Employing It as a Powerful Biocontrol Agent Against Fusarium oxysporum
Source: Int J Mol Sci. 2025 Jan 15;26(2):700. doi: 10.3390/ijms26020700 (PMC11766301; doi:10.3390/ijms26020700)
Supplement: Supplementary file 1 [file ijms-26-00700-s001.zip › ijms-3274639-supplementary.pdf]

SUPPLEMENTAL MATERIALS

Figure S1

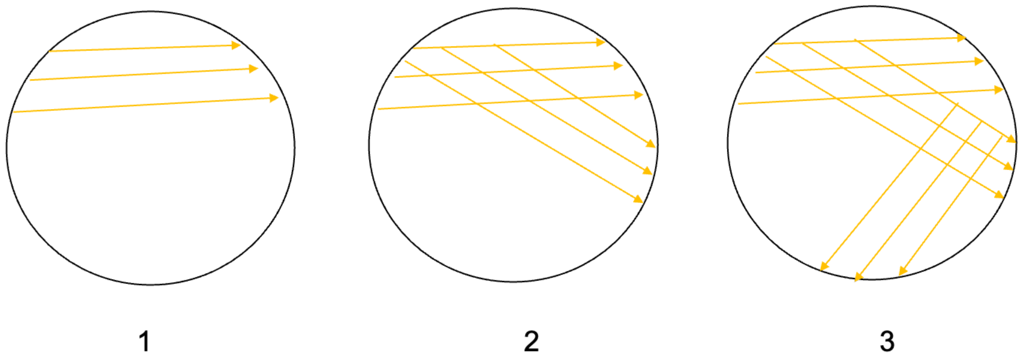

Table S1 Statistical table of assembly result sequence

Sequence ID	Linear/Circular	Sequence Length	GC Content	A Content	T Content	G Content	C Content
Y_4_chr	Circular	3,984,866	46%	1,065,801 (26.75%)	1,068,069 (26.80%)	926,459 (23.25%)	924,537 (23.20%)
Total	-	3,984,866	46%	1,065,801 (26.75%)	1,068,069 (26.80%)	926,459 (23.25%)	924,537 (23.20)
